# Longitudinal profiling of the gut microbiome in patients with psoriatic arthritis and ankylosing spondylitis: a multicentre, prospective, observational study

**DOI:** 10.1186/s41927-020-00155-2

**Published:** 2020-11-10

**Authors:** Jesus Miguens Blanco, Federica Borghese, Neil McHugh, Peter Kelleher, Raj Sengupta, Julian R. Marchesi, Sonya Abraham

**Affiliations:** 1 Division of Digestive Diseases, Department of Metabolism, Digestion and Reproduction, Faculty of Medicine, Imperial College London, London, W2 1NY UK; 2 NIHR/Wellcome Trust Imperial CRF, Imperial Centre for Translational and Experimental Medicine, Imperial College Healthcare NHS Trust, Hammersmith Hospital, Du Cane Road, London, W12 0HS UK; 3 Department of Pharmacy and Pharmacology, University of Bath, Claverton Down, Bath, BA2 7AY UK; 4 Chelsea and Westminster Hospital, Department of Medicine, Imperial College London, London, W2 1NY UK; 5 Royal National Hospital for Rheumatic diseases, Bath, BA1 1RL UK

**Keywords:** Psoriatic arthritis, Microbiome, Ankylosing spondylitis, Metabolomics, Gut-join axis

## Abstract

**Background:**

Psoriasis is a chronic inflammatory disease of the skin affecting 2–3% of UK population. 30% of people affected by psoriasis will develop a distinct form of arthritis within 10 years of the skin condition onset. Although the pathogenesis of psoriatic arthritis is still unknown, there is a genetic predisposition triggered by environmental factors. Limited but convincing evidence link the gut microbiome to psoriatic arthritis. The Microbiome in Psoriatic ARThritis (Mi-PART) study propose is to characterise the microbiome-metabolic interface in patients affected by psoriatic arthritis to deepen our understanding of the pathogenesis of the disease.

**Methods:**

This is a multicentre, prospective, observational study. Psoriatic arthritis (*n* = 65) and ankylosing spondylitis (*n* = 30) patients will be recruited in addition to a control group of healthy volunteers (*n* = 30). Patients eligibility will be evaluated against the Criteria for Psoriatic Arthritis (CASPAR), the Bath Ankylosing Spondylitis Activity Index (BASDAI) and the healthy volunteers who fulfil study inclusion and exclusion criteria.

Information regarding their medical and medication history, demographics, diet and lifestyle will be collected. All the participants in the study will be asked to complete a 7-day food diary, to provide stool samples and to complete quality of life questionnaires. Routine clinical laboratory tests will be performed on blood and urine samples. Patients and healthy volunteers with gastrointestinal symptoms, previous history of cancer, gastrointestinal surgery in the previous 6 months or alcohol abuse will be excluded from the study.

**Discussion:**

The aim of this trial is to characterise the microbiome of psoriatic arthritis patients and to compare it with microbiome of healthy volunteers and of patient with ankylosing spondylitis in order to define if different rheumatologic conditions are associated with characteristic microbiome profiles. Investigating the role of the microbiome in the development of psoriatic arthritis could deepen our understanding of the pathogenesis of the disease and potentially open the way to new therapies.

## Background

Psoriasis is a chronic inflammatory skin disorder that affects 2–3% of UK population [1]. It is characterised by a recurring erythematous papular rash without scars [2]. Psoriasis is associated with several different co-morbidities like a characteristic form of inflammatory arthritis [3]. Clinically, psoriatic arthritis (PsA) is mostly identified by the coexistence of psoriasis and arthritis, however PsA can present different patterns: distal and asymmetric oligoarthritis, symmetric polyarthritis, arthritis mutilans and spondyloarthritis [4]. PsA can induce joints damage quickly with 27% of patients showing irreversible damage in the first year from onset [5].

The pathogenesis of the disease is poorly understood however there is evidence of a genetic component in PsA, it has been demonstrated by studies showing an increased recurrence in first degree relatives [6]. Genes that have been demonstrated associated with an increased risk of developing psoriatic arthritis include HLA-B27, IL 13 and PTPN22, with the strongest association demonstrated for HLA B27 [7]. HLA-B27 is a well-established major risk factor for the development of ankylosing spondylitis (AS), but also 15–20% of PsA patients express HLA-B27 with a stronger association with the axial distribution of the disease [8, 9]. Additional risk factors for psoriatic arthritis include obesity [10], smoking [11], the presence of nail psoriasis and possibly the severity and distribution of psoriasis, some of these factors are linked to the host’s microbiome [12, 13].

In conclusion, several studies have demonstrated the presence of a network at the microbiota-metabolite-immunology level in the human setting of PsA, a chronic inflammatory disease [14, 15].

## Rationale

A growing number of evidence links alteration of the gut microbiome to the development of autoimmunity [16–18]. Studies conducted on animal models of ankylosing spondylitis using HLA-B27 rats have demonstrated the role of microbiome in inducing the disease [19, 20]. Scher et al. have recently identified a different gut microbiota in PsA patients and a concomitant reduction in short chain fatty acids (SCFA) when compared with healthy controls [21]. All these evidences suggest a link between an altered gut microbiome and PsA [12, 22]. Furthermore, there are several recognisable clinical patterns of PsA that overlap with AS and some cases may be even indistinguishable [4]. The study of the gut microbiome of PsA and AS patients brings an opportunity to compare if there is further differences that allow a more precise diagnosis of both diseases.

Thus, PsA is ideally suited to a phenomic approach of studying the mechanisms by which microorganisms and their metabolites affect immunity – investigating the gut and skin microbiome in the setting of inflammatory arthritis. This project should result in potential biomarkers of disease and putative signalling molecules for pharmacological analysis in the future. Additionally, by undertaking a metabolomic approach to the microbiome, novel therapeutic targets could be identified, and treatment approaches stratified according to the individual patients’ genetic and microbiomic drivers of inflammation.

## Study Aims

- To stratify PsA and AS patients according to the disease activity and subtypes based on microbial networks.
- To deepen our understanding of psoriatic arthritis and to identify a panel of biomarkers that would allow stratification of patients according to the disease severity and therapy.
- Phenomic characterisation to determine how the microbiome interact with the host and potentially drive disease.
- To test the genetic predisposition to the development of the disease in association with environmental trigger factors.
- Use phenomic data to develop dietary or pharmaceutical interventions.

## Methods and analysis

### Study design

This is a multicentre, prospective and observational study to collect data and samples from consented males and females of any ethnicity origin aged over 18 years, diagnosed with psoriatic arthritis or ankylosing spondylitis or healthy volunteers from two geographical locations.

- Royal National Hospital For Rheumatic Diseases (RNHRD), Bath, UK.
- Hammersmith Hospital, London, UK.

We will recruit a total of 125 subjects (95 PsA, 30 AS) and 30 healthy volunteers (Table 1). This study adheres to the principles outlined in the NHS Research Governance Framework for Health and Social Care (2nd edition) [23]. It is also conducted in compliance with the Data Protection Act and the Standard Protocol Items: Recommendation for Interventional Trials (SPIRIT) guidelines for clinical studies (Table 2).

**Table 1:** The study is being recruited into four cohorts. The different number of visits is separated by 12 weeks

Group	Health Status	Number of subjects	Number of visits per subject
Group A	Psoriatic Arthritis	15	Screening, visit 1,2 and 3
Group B	Psoriatic Arthritis	50	Screening and visit 1
Group C	Ankylosing Spondylitis	30	Screening and visit 1
Group D	Healthy volunteers	30	Screening and visit 1

**Table 2:** Standard Protocol Items: Recommendation for Interventional Trials (SPIRIT)

	Mi-PART STUDY PERIOD					
	Enrolment	Allocation	Post-allocation			Close-out
**TIMEPOINT(weeks)**	***-2***	**-1**	***0***	***12***	***24***	***T***_***24+***_ ***12 months***
**ENROLMENT**						
**Eligibility screen**	X					
**Informed consent**	X					
**Clinical assessments**		X				
**Allocation**		X				
**ASSESSMENTS**						
***Baseline variables***	X	X	X	X	X	
***Outcome variables***			X	X	X	X
***Data process variables***				X	X	X

### Participants and recruitment

The patients will be recruited from rheumatology clinics under consultant’s advice. Self-referred patients will be involved through advertisements on noticeboards or websites/newspapers. All the questionnaires, indexes and scores mention in this section are detailed in the Additional file 1.

Any male or female patients older than 18 that fulfil the CASPAR or the BASDAI criteria will be invited for screening. They will receive the full patient information sheet (PIS) and informed consent forms (ICF). Potential participants will be allowed to consider full implications of taking part in the study for a minimum of 24 h before screening. They will also have the opportunity to ask questions before signing the consent.

Patients will not be able to take part to the study if they:

- Are not able to give informed consent
- Are pregnant or breastfeeding
- Have a history of alcohol, drugs or chemical abuse in the 3 months prior to screening
- Have recently changed their diet (less than 4 weeks)
- Have not been on a stable therapy with DMARDs in the 3 months prior to enrolment
- Experienced any gastrointestinal symptom (diarrhoea, vomiting, abdominal pain, constipation) in the 4 weeks prior enrolment
- Taken antibiotics in the 3 months prior to enrolment
- Have been diagnosed with cancer in the past 3 years or any primary or secondary immunodeficiency
- Have had endoscopy procedures carried out in the 8 weeks prior to enrolment or any gastro-intestinal surgery in the last 6 months
- Make regular use of laxatives and/or agents that affect gut motility

The healthy volunteers will be approached by the healthy volunteer data manager at Imperial College London and will also able to self-refer through advertisements. After consenting there will be a questionnaire. The volunteer will be asked to complete a self-description of health. This will be evaluated by one of the investigators, and any healthy volunteer with no current medical conditions and not in use of prescribed or self-prescribed medication (except for multivitamins) will be enrolled in the study.

All the participants to the study will receive a physical examination with assessment of vital signs (pulse and blood pressure), weight, height and body mass index (BMI). The medical and medication history will be reviewed with collection of data on demographics, diet and lifestyle. All concomitant medications will be recorded, including probiotics or fermented milk products.

Patients with AS will be evaluated with the Bath Ankylosing Spondylitis Disease Activity Index (BASDAI), the Bath Ankylosing Spondylitis Functional Index (BAFMI) and the Bath Ankylosing Spondylitis Metrology Index (BASMI). Patients with PsA will be evaluated following the CASPAR criteria, the Psoriasis Area and Severity Index (PASI) and the Composite Psoriatic Disease Activity Index (CPDAI). In cases where dactylitis or enthesitis are features, the Leeds Dactylitis Index (LDI) and the Leeds Enthesitis Index (LEI) will be included, respectively. BASDAI, BASMI and BAFMI will be used in cases of PsA with spondyloarthropathy.

All patients will have to complete a Health assessment questionnaire (HAQ), Visual Analogue Scoring (VAS) and a 7-day food diary. PsA patients will have also to complete a Dermatology Life Quality Index Questionnaire (DLQI).

Routine clinical laboratory tests will be performed on all patients other than healthy volunteers, including: full blood count (FBC), liver function tests (LFT), urea and electrolytes (U&E), C-reactive protein (CRP), erythrocyte sedimentation rate (ESR) (London site) / plasma viscosity (PV) (Bath site). Samples will be collected for analysis from all patients: serum, plasma, urine and faeces, as well as DNA assessment.

### Withdrawal criteria

Study participants will be informed that they have the right to withdraw from the study at any time for any reason, without prejudice to their medical care. The investigator can withdraw subjects from the study for any of the following grounds:

1. Subject request.
2. Subject is lost to follow-up (LTFU).
3. Development of an intercurrent illness, condition, or procedural complication, which would interfere with the subject’s continued participation.
4. The investigator also reserves the right to withdraw subjects in the interest of subject safety and welfare.

### Adverse events

#### Definition

##### Adverse Event (AE)

Any untoward medical occurrence in a patient or clinical study subject [24]. This includes occurrences that are not necessarily caused by or related to a medicinal product administered. An adverse event can, therefore, be any unfavourable and unintended signs [25], abnormal laboratory values, and symptoms or disease temporarily associated with or not associated with study activities.

##### Serious Adverse Event (SAE)

Serious Adverse Event. An untoward occurrence that: (a) results in death; (b) is life-threatening; (c) requires hospitalisation or prolongation of existing hospitalisation; (d) results in persistent or significant disability or incapacity; (e) consists of a congenital anomaly or birth defect.

Medical judgement should be exercised in deciding whether an AE is serious in other situations. Important AEs that are not immediately life-threatening or do not result in death or hospitalisation but may jeopardise the subject or may require intervention to prevent one of the other outcomes listed in the definition above, should also be considered serious [26].

#### Reporting procedures

All adverse events should be reported. Depending on the nature of the event the reporting procedures below should be followed. Any questions concerning adverse event reporting should be directed to the Chief Investigator in the first instance.

All such events, whether expected or not, should be recorded. This starts from the signed informed consent for participation in the trial until the last visit.

Adverse events should be documented in the participant’s source document sheet.

Adverse events to be graded according to the degree of severity as follows:

- Grade 1 - Mild, usually transient in nature and does not interfere with normal activity, observation only, no intervention, is needed.
- Grade 2 - Moderate, interfere with normal activities minimal, local or non-invasive intervention indicated; limiting age-appropriate.
- Grade 3 - Severe or medically significant but not immediately life-threatening; hospitalisation or prolongation of hospitalisation indicated; disabling; limiting self-care ADL.
- Grade 4 - Life-threatening consequences; urgent intervention indicated.
- Grade 5 - Death related to AE.

Abnormal laboratory tests results constitute adverse events only if they:

1. Induce clinical signs and symptoms
2. They are considered clinically significant by the investigator
3. They require intervention

Clinically significant abnormal laboratory test results should be identified through a review of Lab values outside normal ranges as per hospital policy and procedures. Notable or significant changes from the baseline or previous visit values, which are considered to be clinically significant, to be documented in the medical notes and reported to their general practitioner and Serious AEs.

An SAE form should be completed and given/sent to the Chief Investigator within 24 h. However, relapse and death due to PsA or AS and hospitalisations for elective treatment of a pre-existing condition do not need reporting as SAEs. All SAEs should be reported to the approving REC where in the opinion of the Chief Investigator, the event was:

1. ‘related’,i.e. resulted from the administration of any of the research procedures; and
2. ‘Unexpected’, i.e. an event that is not an expected occurrence of the study procedures.

Reports of related and unexpected SAEs should be submitted within 15 days of the Chief Investigator becoming aware of the event, using the NRES SAE form for non-IMP studies. The Chief Investigator must also notify the Sponsor of all SAEs.

Local investigators should report any SAEs as required by their Local Research Ethics Committee, Sponsor and/or Research & Development Office.

#### Trial challenge

Emerging evidence has been linking the microbiome with several diseases including autoimmune diseases such as Psoriatic Arthritis [14]. In a paper published in 2015 [21], Scher and colleagues described the observation of a less diverse microbiome in 16 subjects affected by Psoriatic arthritis. Comparing samples taken from patients with psoriatic arthritis, psoriasis and healthy volunteers they observed a lower abundance of specific beneficial taxa of the gut microbiome in the first two groups. Furthermore, the patients with psoriatic arthritis showed other immunologic alterations with increase in fecal sIgA levels, decrease of RANKL and Osteoprogerin (which may affect the antigen presentation process in the gut) and decreased quantities of medium chain fatty acids (MCFAs). From studies conducted on healthy volunteers we know that the microbiome is constantly reshaping under the influence of individual and environmental factors [27–29]. Among the environmental factors, the diet [30, 31] exerts an extremely important role. It has been documented that a change of diet is actually able to modify the microbiome in as little as one or 2 days [32]. In order to detail accurately the relationship between food intake and microbiome, Johnson and colleagues [33] conducted a small clinical trial study on 34 healthy volunteers who were randomised to introduce with different diets. The results of the trial show the potential role of different food and micronutrients in influencing different bacteria strains in a population of healthy volunteers.

The hypothesis of our study was that the microbiome-metabolic interface of psoriatic arthritis patient is distinct comparing to healthy volunteers. The food diary included in the study enlists different elements in the 5 food groups and the possible effect of portion size is taken into account using visual aids to help the patients to describe in the most accurate way the amount of food they have introduced with any meal. The time of the different meals is taken into consideration as are soft and alcoholic drinks.

#### Sample size consideration

Estimate adequate sample sizes is complicated due to the lack of consensus in microbiome studies. Classical power calculations, used for clinical trials, are not a robust manner to assess the numbers of individuals required for exploratory and observational microbiome studies [34] since the SD and means of the sample populations is not known.

The recruitment strategy will be based on previous studies in inflammatory arthropaties [15, 21]. The variance in Th17, Treg numbers, IL-17 and IL − 22 cytokine measurements was estimated on the basis of previous clinical trials results [24]. Assuming a 2-sided significance level of 5% (a = 0.05) and a power 1-b of 95% (level at which we set the null hypothesis) and a 25% difference in IL − 17A secretion between controls and PsA, we needed to recruit a minimum of 24 study participants into each group (healthy volunteer, PsA and AS). Due to the nature of the sampling and the multiple visits to the clinic, we have set the target in 30 volunteers or more per group, to allow for dropout rate of 10% and to ensure robustness. First approach to subject lost to follow-up would be replaced to maximise data outcome and study objectives, otherwise the subject would be included with the acquired data.

In conclusion the study target to enrol 65 eligible patients with psoriatic arthritis (45 at the Bath centre and 20 at the London centre), 30 with ankylosing spondylitis, and 30 healthy volunteers.

### Analysis plan and data management

The phenomic characterisation will take place during the later stage of the study (Table 2) and it involves 3 different arms of analysis: microbiome, immunology and metabonome analysis.

Bacterial DNA will be extracted from faecal samples, to define bacterial diversity and taxonomy. Whole genome sequencing, 16S rRNA gene sequencing and Nanopore sequencing will provide a taxonomic analysis and taxon list in PsA, AS and healthy individuals. Furthermore, this procedure will provide a comparative between the different sequencing technologies and its possibilities in clinical studies.

The immunological analysis will measure proportions of Th17and Treg subsets in peripheral blood samples using multi-parametric flow cytometry. T cells subsets will be use to look for associations between composition of gut microbiome and proportion of circulating Th17 and Treg in study participants. Serum samples will also be used for analysis of pro-inflammatory cytokines (IL-1β, IL-6, and IL-23p19).

NMR and UPLC-MS measurements of all urine, blood and faecal samples for metabolite profiling will be used for the metabonomic characterization. This metabonomic dataset will be used for biomarker ID and functional metabolic discriminators of PsA.

Finally, gut microbiome sequencing datasets will be integrated with biofluid metabolite profiles to identify important biological pathways in specific individuals. In the first stage we will correlate the metabolic profiles from NMR based metabonomics with the pattern of the abundance of microbial taxonomic entities and in a second phase evaluating enrichment of specific ontologies or metabolic functions from metagenomic databases. Once key pathways are statistically pinpointed, we will look into metabolic fluxes as integrated by different taxonomic entities.

The aim is to link gene composition, host metabotypes and immunotypes. We will use the whole gut microbiota community to pinpoint which functions are enriched/depleted in PsA cohorts and whether there are functions which correlate with disease or act as biomarkers of disease.

Meta-gene analysis (i.e. targeted functional metagenomics) will be used to establish the bile salt hydrolases, SCFA synthesizing genes and glucuronidases in gut samples. This approach will link specific functional gene diversity with specific metabotypes.

To ensure that all the data from the study will also be accessible, Mi-PART will upload all his dataset to The European Genome – Phenome Archive [35] and some specific data such as the taxonomy and species diversity of the study will be uploaded to NCBI Taxonomy [36]. Subsequently, data will be made available to the public in the main manuscript or additional supporting files in open access publications.

All data collected during the course of the study will be anonymised with either a temporary I.D. or enrolment I.D and stored at each site in a locked filing cabinet, with restricted access, and disposal arrangements of participant personal and clinical data. Shared data for analysis between centres will be anonymised, password locked and encrypted. Secure platform for sharing data will include email (nhs.net), and other secure media (encrypted USB and CD) via registered mail.

Data and all appropriate documentation will be stored for a minimum of 10 years after the completion of the study, including the follow-up period. The study documentation for the external sites will be transferred and archived at the Imperial College Archives and Corporate Records Unit. Patient identifiable data (PID) will be stored at the respective sites.

Chief Investigator (CI) has a responsibility to ensure that participant anonymity is maintained and protected against any unauthorised parties. Information with regards to study participants will be kept confidential and managed in agreement with the Data Protection Act, NHS Caldicott Guardian, The Research Governance Framework for Health and Social Care and Research Ethics Committee Approval.

To ensure this is done accordingly, each participant at a time of consent will be allocated a unique screening number/code before undergoing any screening procedures. The study management team, together with all clinical site team, will comply with all aspects of the Data Protection Act 1998. All information collected will be kept confidential, and consent forms (containing participants’ full names) will be held entirely separate to another data.

All data collection forms will be anonymised with either a temporary I.D. or enrolment I.D and stored at each site in a locked filing cabinet, with restricted access, and disposal arrangements of participant personal and clinical data.

The anonymised electronic data will be stored and shared on Imperial College Box file storage site. The electronic database used for this study is a bespoke system which uses a robust tried and tested security system with restricted access and routine backup protocols in place.

The anonymised, password locked encrypted data can also be shared between centres using secure email (nhs.net), and other secure media (encrypted USB and CD) via registered mail.

Participants will not be identifiable in the results of the study. The samples collected will also be anonymised with enrollment ID, cohort ID and study visit number. Samples will be kept in the tissue bank at the site of collection until ready for analysis. Anonymised samples that require immediate processing and analysis will be shipped immediately to the designated centres at Imperial College London and Bath University.

Sample collected across sites for processing and analysis will be transferred via registered courier compliant to UN3373 biological substance category B shipment. The participants will be anonymised with regards to any future publications relating to this study.

At the end of the study residual samples will be transferred to a tissue bank**.**

## Discussion

Growing evidence during recent years has linked gut microbiome alterations with an increased disposition to develop chronic rheumatic diseases [15]. Particularly, the PsA link to the microbiome has been the target of a number of promising studies [12, 37–40]. However, there is a paucity of information at the microbiota-metabolite-immunology network level in the setting and development of the disease.

Taking into consideration the gap between these early stages of the research and clinical applications or therapeutic benefits. MI-PART is a multicentre, prospective, and observational study that aims to reduce the gap, undertaking a deep phenomic analysis.

Mi-PART study design acknowledges the heterogeneous manifestations of rheumatic diseases, particularly PsA. Previously, this has led to inaccuracies in the recruitment of patients for clinical studies [41]. All the recruitments in our study fulfil CASPAR Criteria [42] which has been proven the most feasible, specific and sensible for PsA diagnosis [43]. Mi-PART ensures a relevant number of recruitments since it has access to the Axial Disease in Psoriatic Arthritis (ADIPSA) [9] which counts with 200 PsA patients and also 200 AS patients that will set a cohort to analyse the heterogeneous manifestations of PsA and possible overlapping between the two diseases.

Previous studies have reported the relevance of control populations that should be used for microbiome studies [44, 45]. This study will recruit, in the first place, relatives of the PsA patients recruited for the study in order to ensure a similar background. In any circumstance, healthy controls sex, age, ethnicity, etc. will match the features of the PsA cohort to minimise environmental variables, particularly sensible in microbiome studies. Diet of all the participants in the study will be assessed using a 24-h food recall questionnaire for the previous 7 days of every visit during the study.

Diet pivotal role [31, 46, 47] in this study accounts for its connection with gut microbiome dynamics and high plasticity. However, an even bigger challenge is to understand and reconstruct the gut microbiome variability across time, for that reason longitudinal studies are extremely important. To address this, a PsA group in the study will have three different time points with a 12-week separation.

Recent reviews and guidelines for microbiome studies emphasize the need for transition from metataxonomics (16S rRNA gene sequencing) to metagenomics (whole metagenomics shotgun sequencing) since it would allow species-level identification and functional analysis [48–50]. Mi-PART study will run both methodologies in all the samples for a comparison of the cost-effectiveness in clinical studies and potential therapeutic interventions. Furthermore, the study will also use third-generation sequencing using Nanopore sequencing [51] which will give a timely state of the art of the technology and its possibilities for clinical studies and trials.

Even when sequencing technological development has been fundamental in microbiome studies, the importance of the host response to the changes of the microbiome has introduced multi-omics and phenomic analysis as an integrative tool to reveal microbe interaction with the immune and metabolic system [52]. This study would implement a phenomic approach, this work package uniquely interfaces genetics, microbiota, metabolic and inflammatory networks to elucidate the mechanisms driving PsA pathology.

In conclusion, the MI-PART trial is a human microbiome study sets to a full characterization of PsA microbiome and its biomarkers/signalling molecules. The study design, recruitment and sample manipulation have been focus on previous challenges in microbiome and chronic rheumatoid diseases studies, in order to minimize possible confounders and lack of standardization in the study pipeline. Investigating the role of the microbiome in the development of PsA could deepen our understanding of the pathogenesis of the disease and potentially open the way to new therapies.

## Supplementary information

**Additional file 1.** Includes appendices with the different questionnaires use for groups A, B, C & D of the study.

## Data Availability

Not applicable.

## Abbreviations

16S RNA: 16S ribosomal RNA
AS: Ankylosing Spondylitis
BAFMI: Bath Ankylosing Spondylitis Functional Index
BASDAI: Bath Ankylosing Spondylitis Activity index
BASMI: Bath Ankylosing Spondylitis Metrology Index
BMI: Body Mass Index
CASPAR: Criteria for Psoriatic Arthritis
CPDAI: Composite Psoriatic Disease Activity index
CRP: C-Reactive Protein
DLQI: Dermatology Life Quality Index Questionnaire
DMARDs: Disease Modifying Anti-Rheumatic Drugs
DNA: Deoxyribonucleic acid
ESR: Erythrocyte Sedimentation Rate
FBC: Full Blood Count
HAQ: Health assessment questionnaire
HLA-B27: Human Leucocyte Antigen B27
ICF: Informed Consent Form
IL13: Interleukin 13
IL17A: Interleukin 17A
IL22: Interleukin 22
LDI: Leeds Dactylitis Index
LEI: Leeds Enthesitis Index
LFCAs: Long Chain Fatty Acids
LFT: Liver Function Tests
MCFAs: Medium Chain Fatty Acids
PASI: Psoriasis Activity Score index
PID: Patient identifiable Data
PIS: Patient Information Sheet
PsA: Psoriatic Arthritic
PTPN22: Protein Tyrosine Phosphatase Non-Receptor type 22
PV: Plasma Viscosity
qPCR: Quatitative polymerase chain reaction
RANKL: Receptor activator of nuclear factor kappa-Β ligand
RNA: Ribonucleic acid
SCFAs: Small Chain Fatty Acids
sIgA: Secretive Immunoglobuline A
TH17: T helper cell 17
U&E: Urea and Electolytes
VAS: Visual Analogue Scoring
