# Supplementary material for: Longitudinal profiling of the gut microbiome in patients with psoriatic arthritis and ankylosing spondylitis: a multicentre, prospective, observational study
Source: BMC Rheumatol. 2020 Nov 10;4:60. doi: 10.1186/s41927-020-00155-2 (PMC7653819; doi:10.1186/s41927-020-00155-2)
Supplement: Supplementary file 1 — Additional file 1. Includes appendices with the different questionnaires use for groups A, B, C & D of the study. [file 41927_2020_155_MOESM1_ESM.pdf]

APPENDICES

APPENDIX A: Schedule of events and activities

**Table 1. Screening schedule of events and activities**		
**Visit number**		**Screening**<br>(Group A,B,C and D)
**Week**		**Week - 4 to 0**
**Schedule of events and activities**		
**1.**	Written informed consent	**X**
**2.**	Inclusion/exclusion criteria	**X**
**3.**	Demographics & medical history	**X**
**4.**	Concomitant medication	**X**
**5.**	Physical Examination	**X**
**6.**	Research Blood Sampling	**X**
**7.**	Vital signs: Pulse, BP, Height, Weight (kg), BMI.	**X**
**8.**	Adverse events evaluation	**X**
**9.**	CASPAR criteria (Group A&B)	**X**
**10.**	Ankylosing Spondylitis New York Criteria (mNYC) (Group C)	**X**
**11.**	Self-description of health questionnaire (Group D)	**X**

Group A and B

**Table 2.Schedule of events and activities**					
**Visit number**<br>**Group**		**Visit 1**<br>**(A&B)**	**Visit 2**<br>**(A only)**	**Visit 3**<br>**(A only)**	**Early withdrawal**
**Week**		**Week 0**	**Week 12**	**Week 24**	-
Visit window +/-calendar days		+/-30	+/-30	+/-30	-
**Schedule of events and activities**					
**1.**	Concomitant medication since last visit	**X**	**X**	**X**	**X**
**2.**	Adverse events evaluation	**X**	**X**	**X**	**X**
**3.**	Vital signs: Pulse, BP, Height, Weight (kg), BMI	**X**	**X**	**X**	**X**
**4.**	Eligibility check (between screening and baseline only)	**X**			
**5.**	TJC&SJC, PASI, DLQI, HAQ-DI, VAS, CPDAI, LEI, LDI, 7 days food diary, diet and lifestyle questionnaire.	**X**	**X** (A only)	**X** (A only)	**X**
**6.**	BASDAI, BASMI, BAFSI (Only if PsA subtype) spondyloarthropathy)	**X**	**X**	**X**	**X**
**Routine clinical lab investigation**					
**7.**	FBC, LFT, U&E and Creatinine, CRP all sites.<br>ESR (London site) / Plasma Viscosity (Bath site)	**X**	**X**	**X**	**X**
**Study specific lab investigations**					
**8.**	Serum, plasma and DNA sample	**X**	**X**	**X**	**X**
**9.**	Urine and stool samples	**X**	**X**	**X**	**X**

**10.**	**End of study**	(Group B) X		(Group A) X	X

Group C

Table 3. Schedule of events and activities		
**Visit number**		**Visit 1** (Baseline)
**Week**		**Week 0**
Visit window +/-calendar days		+/- 30 days
**Schedule of events and activities**		
**1.**	Concomitant medication	**X**
**2.**	Adverse events evaluation	**X**
**3.**	Vital signs; Pulse, BP, Height, Weight (kg), BMI.	**X**
**4.**	Eligibility check (between screening and baseline)	**X**
**5.**	BASDAI, BASMI, BAFSI, HAQ-DI, VAS, 7 days food dairy, diet and lifestyle questionnaire	**X**
**Routine clinical lab investigation**		
**6.**	FBC, LFT, U&E and Creatinine, CRP and Plasma Viscosity	**X**
**Study specific lab investigations**		
**7.**	Serum, plasma and DNA sample	**X**
**8.**	Urine and stool samples	**X**
**9.**	End of study	**X**

Group D

Table 4. Schedule of events and activities		
**Visit number**		**Visit 1** (Baseline)
**Week**		**Week 0**
	Visit window +/-calendar days	+/ - 30 days
**Schedule of events and activities**		
1.	Concomitant medication	**X**
2.	Adverse events evaluation	**X**
3.	Vital signs: Pulse, BP, Height, Weight (kg), BMI.	**X**
4.	Eligibility check (between screening and baseline)	**X**
**5.**	7days food diary, diet and lifestyle questionnaire	**X**
**Study specific lab investigations**		
6.	Serum, plasma and DNA sample	**X**
7.	Urine and stool samples	**X**
8.	End of study	**X**

APPENDIX B: List of popular fermented milk products and prohibited drugs

**Table 5 List of popular fermented milk products**	
**1.**	KEFIR: (Biona Organic Kefir, Rhythm Coconut Kefir 'one shot' drinks, or Lifeway Frozen Kefir)
**2.**	YOGURT: (Yeo Valley Greek Style natural yoghurt) (CoYo Coconut Milk Yoghurt)
**3.**	Commercial PROBIOTICS: (Yakult, Actimel, Activia)

**Table 6 List of prohibited drugs.**	
**1.**	Domperidone
**2.**	Antibiotics, antifungals, cephalosporins, antivirals, anti-TB drugs

APPENDIX C: List of data to be collected

**Table 7**	**List of data to be collected**			
**Number**	**Group**	**PsA**	**AS**	**HC**
1.	Age			
2.	Gender			
3.	BMI			
4.	ETHNICITY			
5.	DEMOGRAPHICS			
6.	MEDICAL HISTORY			
7.	MEDICATION HISTORY			
8.	CONMEDS			
9.	DISEASE ACTIVITY SCORES			Not required
10.	CASPAR CRITERIA		Not required	
11.	VAS			

12.	DLQI		Not required	
13.	PASI			
14.	HAQ-DI			
15.	LEI			
16.	LDI			
17.	TJC/SJC			
18.	CPDAI			
19.	modified NEW YORK CRITERIA	Not required		
20.	BASMI	Only if subtype		
21.	BASFI			
22.	BASDAI			
23.	HAQ-DI			
24.	Blood pressure			
25.	Pulse			
26.	Weight			
27.	Height			
	**ROUTINE BLOOD SAMPLES**			
28.	U&E and creatinine, LFT, FBC ,CRP all sites.<br>ESR (London), PV (BATH)			
	**STUDY SPECIFIC SAMPLES**			
29.	Serum, plasma and DNA sample			
30.	Stool and Urine			
	**DIET AND HABITS**			
31.	Smoking history			
32.	Alcohol per unit			
33.	7 days food dairy			
34.	Diet Habits and intake of probiotics or fermented milk products			

APPENDIX D: Assessment tools, Quality of life, Lifestyle and Diet health Questionnaires

Date:	D	D	M	M	M	Y	Y	Y	Y
Patient ID:					Visit:				
Centre ID:									

DERMATOLOGY LIFE QUALITY INDEX

DLQI SCORE ☐

The aim of this questionnaire is to measure how much your skin problem has affected your life OVER THE LAST WEEK. Please tick ☑ one box for each question.

**1.**	Over the last week, how **itchy**, **sore, painful** or **stinging** has your skin been?	Very much<br>A lot<br>A little<br>Not at all	☐<br>☐<br>☐<br>☐		
**2.**	Over the last week, how **embarrassed** or **self-conscious** have you been because of your skin?	Very much<br>A lot<br>A little<br>Not at all	☐<br>☐<br>☐<br>☐		
**3.**	Over the last week, how much has your skin interfered with you going **shopping** or looking after your **home** or **garden**?	Very much<br>A lot<br>A little<br>Not at all	☐<br>☐<br>☐<br>☐	Not relevant	☐

Date:	D	D	M	M	M	Y	Y	Y	Y
Patient ID:					Visit:				
Centre ID:									

**4.**	Over the last week, how much has your skin influenced the **clothes** you wear?	Very much<br>A lot<br>A little<br>Not at all	☐<br>☐<br>☐<br>☐	Not relevant	☐
**5.**	Over the last week, how much has your skin affected any **social** or **leisure** activities?	Very much<br>A lot<br>A little<br>Not at all	☐<br>☐<br>☐<br>☐	Not relevant	☐
**6.**	Over the last week, how much has your skin made it difficult for you to do any **sport**?	Very much<br>A lot<br>A little<br>Not at all	☐<br>☐<br>☐<br>☐	Not relevant	☐
**7.**	Over the last week, has your skin prevented you from **working** or **studying**?<br>If "**No**", over the last week how much has your skin been a problem at **work** or **studying**?	Yes<br>No<br>A lot<br>A little<br>Not at all	☐<br>☐<br>☐<br>☐<br>☐	Not relevant	☐
**8.**	Over the last week, how much has your skin created problems your **partner** or	Very much<br>A lot	☐<br>☐		

Date:	D	D	M	M	M	Y	Y	Y	Y
Patient ID:					Visit:			
Centre ID:								

	any of your **close friends** or **relatives**?	A little<br><br>Not at all	☐<br><br>☐	 <br>Not relevant	 <br>☐
**9.**	Over the last week, how much has your skin caused any **sexual difficulties**?	Very much<br>A lot<br>A little<br>Not at all	☐<br>☐<br>☐<br>☐	 <br><br>Not relevant	 <br><br>☐
**10.**	Over the last week, how much of a problem has the **treatment** for your skin been, for example by making your home messy, or by taking up time?	Very much<br>A lot<br>A little<br>Not at all	☐<br>☐<br>☐<br>☐	 <br><br>Not relevant	 <br><br>☐

Please check you have answered EVERY question. Thank you.

© A Y Finlay, G K Khan April 1992 www.dermatology.org.uk, this must not be copied without the permission of the authors.

The scoring of each question is as follows:

Date:	D	D	M	M	M	Y	Y	Y	Y
Patient ID:					Visit:				
Centre ID:									

Response	Score
**Very much**	Scored 3
**A lot**	Scored 2
**A little**	Scored 1
**Not at all**	Scored 0
**Not relevant**	Scored 0
**Question unanswered**	Scored 0
**Question 7 ''prevented work or studying''**	Scored 3

The DLQI is calculated by summing the score of each question resulting in a maximum of 30 and a minimum of 0. The higher the score, the more quality of life is impaired. The DLQI can also be expressed as a percentage of the maximum possible score of 30.

Assessment Reviewed and Verified by:

Name________________________________

Signature____________________________ Date

HEALTH ASSESSMENT QUESTIONNAIRE (HAQ-DI)

Please place an "X" in the box which best describes your abilities OVER THE PAST WEEK:

	WITHOUT ANY DIFFICULTY(0)	WITH SOME DIFFICULTY(1)	WITH MUCH DIFFICULTY(2)	UNABLE TO DO(3)	CATEGORY SCORE
**DRESSING & GROOMING**					
**Are you able to:**					

Imperial College Healthcare NHS Trust | Imperial College London | Mi-PART

Date:	D	D	M	M	M	Y	Y	Y	Y
Patient ID:					Visit:			
Centre ID:								

Dress yourself, including shoelaces and buttons?	☐	☐	☐	☐	☐
Shampoo your hair?	☐	☐	☐	☐	
**ARISING**					
**Are you able to:**					
Stand up from a straight chair?	☐	☐	☐	☐	☐
Get in and out of bed?	☐	☐	☐	☐	
**EATING**					
**Are you able to:**					
Cut your own meat?	☐	☐	☐	☐	☐
Lift a full cup or glass to your mouth?	☐	☐	☐	☐	
Open a new milk carton?	☐	☐	☐	☐	
	**WITHOUT ANY DIFFICULTY(0)**	**WITH SOME DIFFICULTY(1)**	**WITH MUCH DIFFICULTY(2)**	**UNABLE TO DO(3)**	**CATEGORY SCORE**
**WALKING**					
**Are you able to:**					
Walk outdoors on flat ground?	☐	☐	☐	☐	☐

Date:	D	D	M	M	M	Y	Y	Y	Y
Patient ID:					Visit:				
Centre ID:									

Climb up five steps?	☐	☐	☐	☐	
**HYGIENE**					
**Are you able to:**					
Wash and dry your body?	☐	☐	☐	☐	☐
Take a tub bath?	☐	☐	☐	☐	
Get on and off the toilet?	☐	☐	☐	☐	
**REACH**					
**Are you able to:**					
Reach and get down a 5 pound object (such as a bag of sugar)	☐	☐	☐	☐	☐
Bend down to pick up clothing	☐	☐	☐	☐	
**GRIP**					
**Are you able to:**					
Open car doors?	☐	☐	☐	☐	☐
Open previously opened jars?	☐	☐	☐	☐	
Turn taps on and off?	☐	☐	☐	☐	
**ACTIVITIES**					
**Are you able to:**					
Run errands and shop?	☐	☐	☐	☐	☐
Get in and out of a car?	☐	☐	☐	☐	

Date:	D	D	M	M	M	Y	Y	Y	Y
Patient ID:					Visit:				
Centre ID:									

Do chores such as vacuuming or yard work?	☐	☐	☐	☐	

Please check any AIDS OR DEVICES that you usually use for any of the above activities:

☐	Raised toilet seat	☐	Bathtub bar	☐	Long-handled appliances for reach
☐	Bathtub seat	☐	Long-handled appliances in bathroom	☐	Jar opener (for jars previously opened)

Please check any categories for which you usually need HELP FROM ANOTHER PERSON:

☐	Hygiene	☐	Reach	☐	Gripping and opening things	☐	Errands and chores

Once all questions have been completed by the patient the score is added up and divided by eight, giving the overall HAQ SCORE

TOTAL SCORE ☐ **HAQ SCORE** ☐

Date:	D	D	M	M	M	Y	Y	Y	Y
Patient ID:					Visit:				
Centre ID:									

Your ACTIVITIES: To what extent are you able to carry out your everyday physical activities such as walking, climbing stairs, carrying groceries, or moving a chair?

COMPLETELY	MOSTLY	MODERATELY	A LITTLE	NOT AT ALL
☐	☐	☐	☐	☐

Assessment Reviewed and Verified by:

Name________________________________

Signature_____________________________ Date:

Date:	D	D	M	M	M	Y	Y	Y	Y
Patient ID:					Visit:				
Centre ID:									

PSORIASIS AREA AND SEVERITY INDEX (PASI)

1 percentage of Body Surface Area (BSA) is equivalent of patient's one Handprint

**HEAD = 10% OR 0.1 OF TOTAL BSA**					
**SEVERITY** of Psoriatic lesions					
**LESION SCORE** (please circle one)	**NONE**	**MILD**	**MODERATE**	**SEVERE**	**VERY SEVERE**
Induration (thickness)	**0**	**1**	**2**	**3**	**4**
Desquamation (scaling)	**0**	**1**	**2**	**3**	**4**
Erythema (redness)	**0**	**1**	**2**	**3**	**4**
**Total Severity Score=**					

**AREA** affected by Psoriasis							
**Body Surface Area** (BSA)	**0%**	**1-9%**	**10-29%**	**30-49%**	**50-69%**	**70-89%**	**90-100%**
**Equivalent grade Score** (please circle one)	**0**	**1**	**2**	**3**	**4**	**5**	**6**
**TOTAL = Severity Score_______x Area Score________ x 0.1 ____________**							

Date:	D	D	M	M	M	Y	Y	Y	Y
Patient ID:					Visit:				
Centre ID:									

UPPER LIMBS =20% OR 0.2 OF TOTAL BSA					
**SEVERITY** of Psoriatic lesions					
**LESION SCORE** (please circle one)	**NONE**	**MILD**	**MODERATE**	**SEVERE**	**VERY SEVERE**
Induration (thickness)	**0**	**1**	**2**	**3**	**4**
Desquamation (scaling)	**0**	**1**	**2**	**3**	**4**
Erythema (redness)	**0**	**1**	**2**	**3**	**4**
**Total Severity Score=**					

**AREA** affected by Psoriasis							
**Body Surface Area** (BSA)	**0%**	**1-9%**	**10-29%**	**30-49%**	**50-69%**	**70-89%**	**90-100%**
**Equivalent grade Score** (please circle one)	**0**	**1**	**2**	**3**	**4**	**5**	**6**

TOTAL = Severity Score________x Area Score_____x 0.2 =__________

Date:	D	D	M	M	M	Y	Y	Y	Y
Patient ID:					Visit:				
Centre ID:									

PSORIASIS AREA AND SEVERITY INDEX (PASI)

1 percentage of Body Surface Area (BSA) is equivalent of patient's one Handprint

**TRUNK = 30% 0R 0.3 OF TOTAL BSA**					
**SEVERITY** of Psoriatic lesions					
**LESION SCORE** (please circle one)	**NONE**	**MILD**	**MODERATE**	**SEVERE**	**VERY SEVERE**
Induration (thickness)	**0**	**1**	**2**	**3**	**4**
Desquamation (scaling)	**0**	**1**	**2**	**3**	**4**
Erythema (redness)	**0**	**1**	**2**	**3**	**4**
**Total Severity Score=**					

**AREA** affected by Psoriasis							
**Body Surface Area** (BSA)	**0%**	**1-9%**	**10-29%**	**30-49%**	**50-69%**	**70-89%**	**90-100%**
**Equivalent grade Score** (please circle one)	**0**	**1**	**2**	**3**	**4**	**5**	**6**
**TOTAL = Severity Score______x Area Score______x 0.3 =___________**							

Date:	D	D	M	M	M	Y	Y	Y	Y
Patient ID:					Visit:				
Centre ID:									

LOWER LIMBS = 40% OR 0.4 OF TOTAL BSA					
**SEVERITY** of Psoriatic lesions					
**LESION SCORE** (please circle one)	**NONE**	**MILD**	**MODERATE**	**SEVERE**	**VERY SEVERE**
Induration (thickness)	**0**	**1**	**2**	**3**	**4**
Desquamation (scaling)	**0**	**1**	**2**	**3**	**4**
Erythema (redness)	**0**	**1**	**2**	**3**	**4**
**Total Severity Score=**					

**AREA** affected by Psoriasis							
**Body Surface Area** (BSA)	**0%**	**1-9%**	**10-29%**	**30-49%**	**50-69%**	**70-89%**	**90-100%**
**Equivalent grade Score** (please circle one)	**0**	**1**	**2**	**3**	**4**	**5**	**6**

TOTAL = Severity Score________x Area Score_______x 0.4 ____________

Date:	D	D	M	M	M	Y	Y	Y	Y
Patient ID:					Visit:				
Centre ID:									

PSORIASIS AREA AND SEVERITY INDEX (PASI)

TOTAL PASI SCORE	
HEAD	
UPPER LIMBS	
TRUNK	
UPPER LIMBS	
**PASI SCORE=**	

Assessment Reviewed and Verified by:

Name________________________________

Signature_____________________________ Date

Date:	D	D	M	M	M	Y	Y	Y	Y
Patient ID:					Visit:				
Centre ID:									

PSORIATIC ARTHRITIS SWOLLEN AND TENDER JOINT COUNT

Which joints are **TENDER**? (please tick)	Which joints are **SWOLLEN**? (please tick)

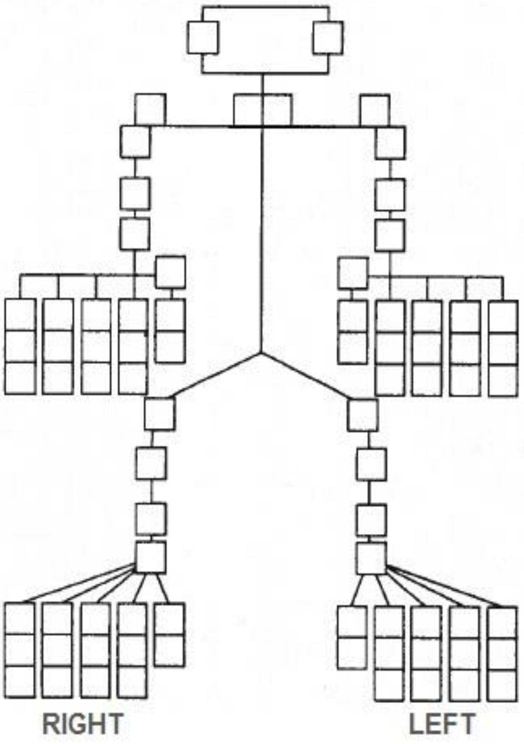

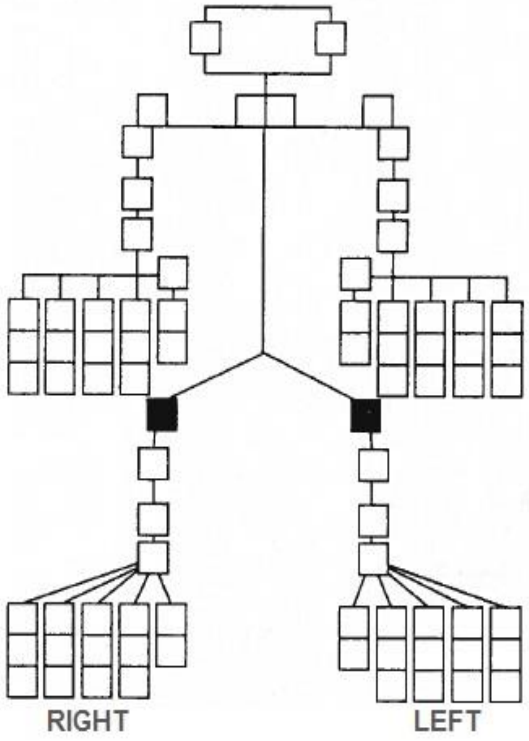

**Tender Joint Count________/68____**	**Swollen Joint Count_______/66_______**

Assessment Reviewed and Verified by:

Name________________________________

Date:	D	D	M	M	M	Y	Y	Y	Y
Patient ID:					Visit:				
Centre ID:									

Signature___________________________ Date

Date:	D	D	M	M	M	Y	Y	Y	Y
Patient ID:					Visit:				
Centre ID:									

VISUAL ANALOGUE SCORING (VAS)

PATIENT GLOBAL HEALTH INDEX

How do you feel concerning your arthritis over the last week?

(*Mark a line on the scale below at the point that best describes how active your arthritis was last week*)

VERY WELL<br>0	(Vas line/100mm)	EXTREMELY BAD<br>100	Score

PATIENT PAIN VISUAL ANALOQUE SCALE

No pain<br>0	(Vas line/100mm)	Worst imaginable pain<br>100	Score

Assessment Reviewed and Verified by:

Name ____________________________

Signature ____________________________ Date

Date:	D	D	M	M	M	Y	Y	Y	Y
Patient ID:					Visit:				
Centre ID:									

Leeds Enthesitis Index (LEI)

The LEI score range is 0-6.

**Leeds Enthesitis Index**	**Left**	**Right**
Lateral epicondyle of elbow	☐	☐
Medical condyle of femur	☐	☐
Achilles tendon insertion	☐	☐
LEI	______________________________	

Assessment Reviewed and Verified by:

Name ________________________________

Signature ____________________________ Date

Date:	D	D	M	M	M	Y	Y	Y	Y
Patient ID:					Visit:				
Centre ID:									

Leeds Dactylitis Index (LDI)

Score range is 0-20: ☐

In the table below, please tick if the digit is affected by dactylitis
If affected, please also tick 'Yes' or 'No' to confirm whether the digit is tender

**Left Fingers** *(Tick if affected by dactylitis)*	**If affected, tender?**		**Right Fingers** *(Tick if affected by dactylitis)*	**If affected, tender?**	
	**Yes**	**No**		**Yes**	**No**
☐ 1. Thumb	☐	☐	☐ 1. Thumb	☐	☐
☐ 2. Index	☐	☐	☐ 2. Index	☐	☐
☐ 3. Middle	☐	☐	☐ 3. Middle	☐	☐
☐ 4. Ring	☐	☐	☐ 4. Ring	☐	☐
☐ 5. Little	☐	☐	☐ 5. Little	☐	☐

**Left Toes** *(Tick if affected by dactylitis)*	**If affected, tender?**		**Right Toes** *(Tick if affected by dactylitis)*	**If affected, tender?**	
	**Yes**	**No**		**Yes**	**No**
☐ 1. Great	☐	☐	☐ 1. Great	☐	☐
☐ 2. Second	☐	☐	☐ 2. Second	☐	☐
☐ 3. Middle	☐	☐	☐ 3. Middle	☐	☐
☐ 4. Fourth	☐	☐	☐ 4. Fourth	☐	☐
☐ 5. Little	☐	☐	☐ 5. Little	☐	☐

Assessment Reviewed and Verified by:

Name ______________________

Date:	D	D	M	M	M	Y	Y	Y	Y
Patient ID:					Visit:				
Centre ID:									

Signature ______________________ Date

Date:	D	D	M	M	M	Y	Y	Y	Y
Patient ID:					Visit:				
Centre ID:									

Composite Psoriatic Disease Activity Index (0 -15)

CPDAI Score:

	None (0)	Mild (1)	Moderate (2)	Severe (3)
**Peripheral Arthritis**	**NONE**	**≤ 4 joints; normal function (HAQ ≤0.5)**	**≤ 4 joints but function impaired; or > 4 joints, normal function**	**> 4 joints and function impaired**
**Skin Disease**	**NONE**	**PASI ≤ 10 and DLQI ≤ 10**	**PASI ≤ 10 but DLQI >10; or PASI > 10 but DLQI ≤ 10**	**PASI > 10 and DLQI > 10**
**Enthesitis**	**NONE**	**≤ 3 sites; normal function (HAQ ≤0.5)**	**≤ 3 sites but function impaired; or >3 sites but normal function**	**>3 sites and function impaired**
**Dactylitis**	**NONE**	**≤ 3 digits; normal function (HAQ ≤0.5))**	**≤ 3 digits but function impaired; or >3 digits but normal function**	**>3 digits and has function impaired**
**Spinal Disease**	**NONE**	**BASDAI ≤4; normal function (ASQol ≤ 6)**	**BASDAI >4 but normal function; BASDAI ≤4 but function impaired**	**BASDAI >4 and function impaired**

❖ CPDAI and treatment implications

	Total Score	Treatment
Mild	0-<5*	Symptomatic
Moderate	5-6**	+ DMARD
Severe	>6	+ anti-TNF

HAQ only counted for most severe domain involved (enthesitis/dactylitis/peripheral arthritis)

Assessment Reviewed and Verified by:

Name ___________________________

Date:	D	D	M	M	M	Y	Y	Y	Y
Patient ID:						Visit:			
Centre ID:									

Signature ______________________ Date

Date:	D	D	M	M	M	Y	Y	Y	Y
Patient ID:					Visit:				
Centre ID:									

The modified New York Criteria (mNYC)

To aid diagnosis of Ankylosing Spondylitis

According to the mNYC for diagnosis of AS, a definite diagnosis of AS requires the patient to satisfy the radiological criterion plus at least one clinical criterion as defined below:

1. **Radiological criterion**

   Sacroilitis at least:

   (i) Grade ≥2 bilaterally or — Yes ☐ No ☐

   (ii) Grade 3-4 unilaterally — Yes ☐ No ☐

2. **Clinical criteria**

   (i) Low back pain and stiffness for more than 3 months that improves with exercise but is not relieved by rest — Yes ☐ No ☐

   (ii) Limitation of the lumbar spine in both the sagittal and frontal planes range of movement — Yes ☐ No ☐

   (iii) Limitation of chest expansion relative to normal values corrected for age and sex — Yes ☐ No ☐

Normal range of movement

- **Sagittal plane (lumbar flexion)[2] is ≥ 7cm**
- **Frontal plane (side flexion)[2] is ≥ 20cm**
- **Chest expansion (Mean cm)[3]**

- **Age 18-24: Male 7.0 cm; Female 5.5 cm**
- **Age 25-34: Male 7.5 cm; Female 5.5 cm**
- **Age 35-44: Male 6.5 cm; Female 4.5 cm**
- **Age 45-54: Male 6.0 cm; Female 5.0 cm**
- **Age 55-64: Male 5.5 cm; Female 4.0 cm**
- **Age 65-74: Male 4.0 cm; Female 4.0 cm**
- **Age 75+: Male 3.0 cm; Female 2.5 cm**

Date:	D	D	M	M	M	Y	Y	Y	Y
Patient ID:						Visit:			
Centre ID:									

Does this patient have AS as measured by the mNYC? Yes ☐ No ☐

Assessment Reviewed and Verified by:

Name ________________________

Signature _______________________ Date

Date:	D	D	M	M	M	Y	Y	Y	Y
Patient ID:					Visit:				
Centre ID:									

Bath Ankylosing Spondylitis Disease Activity Index

BASDAI Score: ☐

Please place an "X" in the box to indicate your answer to each question relating to the past week.

1. How would you describe the overall level of fatigue/tiredness you have experienced?

0	1	2	3	4	5	6	7	8	9	10
☐	☐	☐	☐	☐	☐	☐	☐	☐	☐	☐

None **very severe**

2. How would you describe the overall level of neck, back or hip pain you have had?

0	1	2	3	4	5	6	7	8	9	10
☐	☐	☐	☐	☐	☐	☐	☐	☐	☐	☐

None **very severe**

3. How would you describe the overall level of pain/swelling in joints other than neck, back of hips you have had?

0	1	2	3	4	5	6	7	8	9	10
☐	☐	☐	☐	☐	☐	☐	☐	☐	☐	☐

None **very severe**

Date:	D	D	M	M	M	Y	Y	Y	Y
Patient ID:					Visit:				
Centre ID:									

4. How would you describe the overall level of discomfort you have had from any areas tender to touch or pressure?

0	1	2	3	4	5	6	7	8	9	10
☐	☐	☐	☐	☐	☐	☐	☐	☐	☐	☐

None **very severe**

5. How would you describe the overall level of morning stiffness you have had from the time you wake up?

0	1	2	3	4	5	6	7	8	9	10
☐	☐	☐	☐	☐	☐	☐	☐	☐	☐	☐

None **very severe**

6. How long does your morning stiffness last from the time you wake up?

0	1	2	3	4	5	6	7	8	9	10
☐	☐	☐	☐	☐	☐	☐	☐	☐	☐	☐

0hr **1hr** **2 or more hrs**

Date:	D	D	M	M	M	Y	Y	Y	Y
Patient ID:						Visit:			
Centre ID:									

Assessment Reviewed and Verified by:

Name ___________________________

Signature _______________________ Date

Date:	D	D	M	M	M	Y	Y	Y	Y
Patient ID:					Visit:				
Centre ID:									

Bath Ankylosing Spondylitis Functional Index*

BASFI Score: ☐

*Calin et al. J Rheumatol 1994 21; 2281-85

Please draw a mark on each line below to indicate your ability with each of the following activities, during the past week:

1. Putting on your socks or tights without help or aids (e.g. sock aids)?

**0**	1	2	3	4	5	6	7	8	9	10
☐	☐	☐	☐	☐	☐	☐	☐	☐	☐	☐

2. Bending forward from the waist to pick up a pen from the floor without an aid?

**0**	1	2	3	4	5	6	7	8	9	10
☐	☐	☐	☐	☐	☐	☐	☐	☐	☐	☐

3. Reaching up to a high shelf without help or aids (e.g. helping hand)?

**0**	1	2	3	4	5	6	7	8	9	10
☐	☐	☐	☐	☐	☐	☐	☐	☐	☐	☐

4. Getting up out of an armless dining room chair without using your hands or any other help?

**0**	1	2	3	4	5	6	7	8	9	10
☐	☐	☐	☐	☐	☐	☐	☐	☐	☐	☐

5. Getting up off the floor without any help from lying on your back?

Date:	D	D	M	M	M	Y	Y	Y	Y
Patient ID:					Visit:				
Centre ID:									

**0**	1	2	3	4	5	6	7	8	9	10
☐	☐	☐	☐	☐	☐	☐	☐	☐	☐	☐

6. Standing unsupported for 10 minutes without discomfort?

**0**	1	2	3	4	5	6	7	8	9	10
☐	☐	☐	☐	☐	☐	☐	☐	☐	☐	☐

7. Climbing 12-15 steps without using a handrail or walking aid (one foot on each step)?

**0**	1	2	3	4	5	6	7	8	9	10
☐	☐	☐	☐	☐	☐	☐	☐	☐	☐	☐

8. Looking over your shoulder without turning your body?

**0**	1	2	3	4	5	6	7	8	9	10
☐	☐	☐	☐	☐	☐	☐	☐	☐	☐	☐

9. Doing physically demanding activities (e.g. physiotherapy exercises, gardening or sports)?

**0**	1	2	3	4	5	6	7	8	9	10
☐	☐	☐	☐	☐	☐	☐	☐	☐	☐	☐

10. Doing a full day activities whether it be at home or work?

**0**	1	2	3	4	5	6	7	8	9	10
☐	☐	☐	☐	☐	☐	☐	☐	☐	☐	☐

Date:	D	D	M	M	M	Y	Y	Y	Y
Patient ID:					Visit:				
Centre ID:									

Assessment Reviewed and Verified by:

Name ______________________

Signature ___________________ Date

Date:	D	D	M	M	M	Y	Y	Y	Y
Patient ID:					Visit:				
Centre ID:									

The Bath Ankylosing Spondylitis Metrology Index*

(BASMI) *Jenkinson et al, 1994)

	0	1	2	3	4	5	6	7	8	9	10
**Tragus to wall (cm)**	≤ 10	10–12.9	13–15.9	16-18.9	19–21.9	22-24.9	25-27.9	28-30.9	31-33.9	34-36.9	≥ 37
**Lumbar Flexion (cm)**	≥ 7.0	6.4-7.0	5.7-6.3	5.0-5.6	4.3-4.9	3.6-4.2	2.9-3.5	2.2-2.8	1.5-2.1	0.8-1.4	≤ 0.7
**Intermalleolar distance (cm)**	≥ 120	110-119.9	100-109.9	90-99.9	80-89.9	70-79.9	60-69.9	50-59.9	40-49.9	30-39.9	≤ 30
**Cervical Rotation (degrees)**	≥ 85	76.6-85	68.1-76.5	59.6-68	51.1-59.5	42.6-51	34.1-42.5	25.6-34	17.1-25.5	8.6-17	≤ 8.5
**Lumbar Side Flexion (cm)**	≥ 20	18- 20	15.9-17.9	13.8-15.8	11.7-13.7	9.6-11.6	7.5-9.5	5.4-7.4	3.3-5.3	1.2-3.2	≤ 1.2

**Assessment**	**Measurement**		**Mean**	**BASMI score**
	**Left**	**Right**		
**Tragus to wall distance**				
**Lumbar side flexion**				

Date:	D	D	M	M	M	Y	Y	Y	Y
Patient ID:					Visit:			
Centre ID:								

**Cervical spine rotation**				

**Lumbar spine flexion (modified schober's index)**		**N/A**	
**Intermalleolar distance**		**N/A**	
**Other**		**N/A**	
		**Total score**	
	**BASMI score =**	**Total** = **5**	

Assessment Reviewed and Verified by:

Name ____________________

Signature __________________ Date

Date:	D	D	M	M	M	Y	Y	Y	Y
Patient ID:					Visit:				
Centre ID:									

Healthy Control self-description of health

1. Have you been diagnosed or suspected or investigated for inflammatory bowel diseases (Ulcerative colitis and Crohn's disease) [26]?

   Yes ☐ No ☐

2. In the last four weeks have you experienced any of the following symptoms?
   a. Pain, swelling or cramping in the tummy.
   b. Recurring or bloody diarrhoea
   c. Weight loss without trying.
   d. Extreme tiredness.
   e. None of the above

3. Have you been diagnosed, suspected or investigated for irritable bowel syndrome (IBS)?

   Yes ☐ No ☐

4. In the last four weeks have experienced any of the following symptoms [25]?
   a. Abdominal (stomach) pain and cramping, which may be relieved by having a poo.
   b. A change in your bowel habits – such as diarrhoea, constipation, or sometimes both bloating and swelling of your stomach.
   c. Excessive wind (flatulence).
   d. Occasionally experiencing an urgent need to go to the toilet
   e. A feeling that you have not fully emptied your bowels after going to the toilet
   f. Passing mucus from your bottom.
   g. None of the above

5. Have you undergone gastro-intestinal surgery in the last 6months or presently have gastric band in Place?

Date:	D	D	M	M	M	Y	Y	Y	Y
Patient ID:					Visit:				
Centre ID:									

Yes ☐ No ☐

6. Have you been diagnosed with or suspected to suffer from any medical condition that courses immunodeficiency or immunosuppression?

Yes ☐ No ☐

7. Have you been under a cancer specialist in the last year?

Yes ☐ No ☐

8. Have you been diagnosed with any other medical problems? e.g. Asthma, Hypertension, Diabetes or over/under active thyroid etc.

Yes ☐ No ☐

If yes list here: Mark with "X" to indicate if disease is active or not (not on treatment)

Number	Medical history	Date	Active	Not active
1.		Start __/___/____ stop	☐	☐
2.		Start __/___/____ stop	☐	☐
3.		Start __/___/____ stop	☐	☐
4.		Start __/___/____ stop	☐	☐
5.		Start __/___/____ stop	☐	☐

9. Have you recently or presently taking any medication (prescription or Over the counter)?

Yes ☐ No ☐

Date:	D	D	M	M	M	Y	Y	Y	Y
Patient ID:					Visit:				
Centre ID:									

If yes list the name(s) or class of medication taken e.g. antihypertensive, antacid etc.

Number	Drug name or classification	Start date	Ongoing	Stop Date
1.		__/___/____	☐	__/___/____
2.		__/___/____	☐	__/___/____
3.		__/___/____	☐	__/___/____
4.		__/___/____	☐	__/___/____
5.		__/___/____	☐	__/___/____
6.		__/___/____	☐	__/___/____
7.		__/___/____	☐	__/___/____
8.		__/___/____	☐	__/___/____

Assessment completed by:

Name ________________________________

Signature _____________________________ Date

Date:	D	D	M	M	M	Y	Y	Y	Y
Patient ID:					Visit:				
Centre ID:									
